# Optimism in adults born preterm: Systematic review and individual-participant-data meta-analysis

**DOI:** 10.1371/journal.pone.0259463

**Published:** 2021-11-18

**Authors:** Rachel K. Robinson, Kati Heinonen, Polina Girchenko, Marius Lahti-Pulkkinen, Eero Kajantie, Petteri Hovi, Aulikki Lano, Sture Andersson, Johan G. Eriksson, Dieter Wolke, Sakari Lemola, Katri Räikkönen

**Affiliations:** 1 Department of Psychology & Logopedics, Faculty of Medicine, University of Helsinki, Helsinki, Finland; 2 Welfare Sciences/Psychology, Faculty of Social Sciences, Tampere University, Tampere, Finland; 3 National Institute of Health and Welfare, Helsinki, Finland; 4 University/British Heart Foundation Centre for Cardiovascular Science, Queen’s Medical Research Institute, University of Edinburgh, Edinburgh, United Kingdom; 5 PEDEGO Research Unit, Medical Research Center Oulu, Oulu University Hospital and University of Oulu Finland, Oulu, Finland; 6 Children’s Hospital, Helsinki University Hospital and the University of Helsinki, Helsinki, Finland; 7 Department of Clinical and Molecular Medicine, Norwegian University of Science and Technology, Trondheim, Norway; 8 Department of General Practice Primary Health Care, University of Helsinki, Helsinki, Finland; 9 Folkhälsan Research Center, Helsinki, Finland; 10 Singapore Institute for Clinical Sciences, Agency for Science, Technology and Research, Singapore, Singapore; 11 Department of Obstetrics & Gynecology, National University of Singapore, Singapore, Singapore; 12 Mental Health and Wellbeing, Warwick Medical School, University of Warwick, Coventry, United Kingdom; 13 Department of Psychology, University of Warwick, Coventry, United Kingdom; 14 Department of Psychology, Bielefeld University, Bielefeld, Germany; University of Mississippi Medical Center, UNITED STATES

## Abstract

**Aim:**

Preterm birth(<37 gestational weeks) is associated with numerous adversities, however, data on positive developmental outcomes remain limited. We examined if preterm and term born(≥37 gestational weeks) adults differ in dispositional optimism/pessimism, a personality trait associated with health and wellbeing. We assessed if birth weight z-score, neurosensory impairments and parental education modified the outcome.

**Methods:**

We systematically searched PubMed and Web of Science for cohort or case-control studies(born ≥ 1970) with data on gestational age and optimism/pessimism reported using the Life-Orientation-Test-Revised in adulthood(≥18 years). The three identified studies(Helsinki Study of Very Low Birth Weight Adults; Arvo Ylppö Longitudinal Study; Avon Longitudinal Study of Parents and Children) provided data for the two-step random-effects linear regression Individual-Participant-Data meta-analysis.

**Results:**

Preterm and term borns did not differ on optimism(p = 0.76). Preterms scored higher on pessimism than term borns(Mean difference = 0.35, 95%Confidence Interval 0.36, 0.60, p = 0.007), although not after full adjustment. Preterm born participants, but not term born participants, with higher birth weight z-score, had higher optimism scores (0.30 raw score units per standard deviation increase, 95% CI 0.10, 0.49, p = 0.003); preterm vs term x birth weight z-score interaction p = 0.004).

**Conclusions:**

Preterm and term born adults display similar optimism. In preterms, higher birth weight may foster developmental trajectories promoting more optimistic life orientations.

## Introduction

Worldwide approximately 15 million preterm (<37 gestational weeks) births occur annually, equating to 1 in every 10 deliveries [1]. A major cause of global perinatal mortality and morbidity [2], preterm birth also increases the long term risks of physical [3, 4], mental [5], socioemotional [6], and cognitive [7] problems as well as socioeconomic-related adversities [8–11].

While evidence continues to mount on the negative outcomes following preterm birth, data remains limited on positive developmental outcomes—leaving a disproportionate picture as not all preterms experience adversities. One positive developmental outcome may be dispositional optimism [12, 13]. Individuals high on optimism hold generally positive expectations regarding their future life outcomes, whereas individuals high on pessimism hold negative ones [14]. Previous studies associate higher optimism with better physical health [15], exceptional longevity [16], and decreased risk of mortality [17, 18]. A comprehensive meta-analysis found a significant positive association between overall optimism/pessimism with aggregate positive physical health outcome measures [19]. Optimism has also been associated with lower rates of binge-eating [20]), better health-related quality of life [21], more positive daily mood [22, 23] and less suicidal ideation [24]. Furthermore, studies also indicate that optimists may recover faster or cope better with both physical and emotional stress [25, 26].

### Rationale

To our knowledge, no study has explored if preterm and term born adults differ in dispositional optimism and pessimism. Hence, we systematically reviewed the literature and explored in an individual participant meta-analysis (IPD-MA), if young adults born preterm and at term differed in self-reported dispositional optimism and pessimism. Additionally, we also examined if the differences between preterm and term born adults in optimism and pessimism varied by birth weight z-score, neurosensory impairment, and parental education, because they have been identified as effect modifiers in previous literature on preterm birth [27, 28]. As preterm young adults have been shown to display adversities in health and wellbeing, we hypothesized that preterms would display lower optimism and higher pessimism than term borns. We also predicted that optimism would decrease and that pessimism would increase according to the degree of prematurity. However, importantly, as not all preterms experience adversities, we hypothesized that preterms born with higher birth weight z-scores, without neurosensory impairments and with higher parental education would display higher optimism and lower pessimism.

## Materials and methods

### Protocol & registration

No protocol has been published.

### Eligibility criteria

Cohort and longitudinal case-control studies with published or unpublished data beginning at birth or before, which had measured optimism/pessimism using the Life Orientation Test-Revised (LOT-R) in young adulthood (18–30 years), and had any gestational age data. Study populations born before 1970 were excluded due to the major changes in neonatal medicine.

#### Identification of studies: Search & information sources

The original electronic search was performed in PubMed and Web of Science (inception to 2019) and an updated search was performed in June 2021 in PubMed, Web of Science, ProQuest and EBSCO/Host. The full search strategies are provided in S1 File. The study also collaborated with three major international preterm birth study networks where the availability of unpublished data could, also be assessed; PremLife [29], RECAP [30], APIC [3, 31–33], encompassing a wide breadth of preterm birth, long-term cohort studies in Europe, the Americas, New Zealand and Australia.

#### Selection of studies

**Fig 1:**
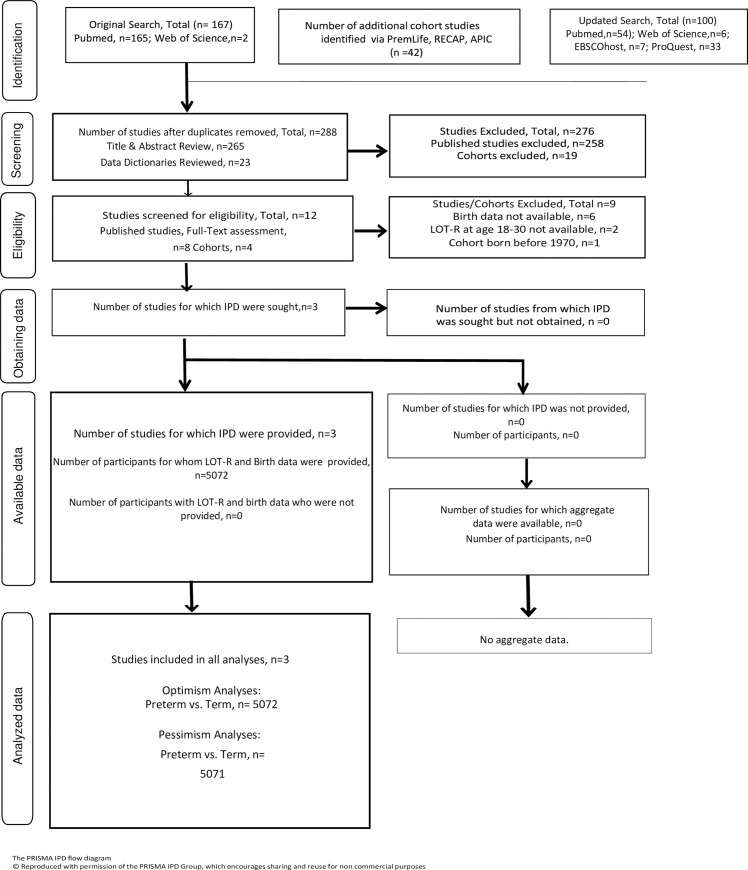
PRISMA flow diagram.

Study selection from the electronic searches occurred in three rounds; 1) Title review of all results for relevance, 2) Abstract review of identified studies for inclusion/exclusion criteria, and 3) Full-text review for final inclusion (Fig 1). For unpublished data, the preterm birth cohort network studies were reviewed for follow-up time points in adulthood, followed by data dictionary review to determine final inclusion based on same inclusion criteria set out for published studies. The Avon Longitudinal Study of Parents and Children (ALSPAC) study website contains details of all the data that are available through a fully searchable data dictionary and variable search tool [34]. All other data dictionaries were provided by partners from the preterm birth study networks.

#### Ethical considerations

The authors assert that all procedures contributing to this work comply with the ethical standards of the relevant national and institutional committee on human experimentation with the Helsinki Declaration of 1975, as revised in 2008. All study protocols were approved by the respective ethics committee of each participating institution and informed consents were obtained from all participants and their parents/guardian.

### Data collection

We included all studies that met the inclusion criteria: ALSPAC [35, 36], the Arvo Ylppö Longitudinal Study(AYLS) [38] and the Helsinki Study of Very low birth weight Adults (HeSVA) [4, 28, 41, 39–51). Data dictionary review identified all necessary variables from each included study. All included studies received an itemized variable level data request with the proposed analysis plan. All missing and non-standard values (e.g. -9999, -11) were recoded as system missing. Data harmonization was performed in SPSS(RR) and was reviewed in duplicate (PG).

### Specification of outcomes and effect measures

All measures for exposure, outcomes and covariates and moderators were pre-specified at the onset of the study. All included studies provided the requested LOT-R, gestational age, birth weight, perinatal and adult follow-up variables outlined below.

#### Optimism and pessimism outcome measurements

Item level data for the participants’ LOT-R [14] measuring dispositional optimism/pessimism were requested and received from each cohort. Participants responded to optimism and pessimism items on a 5-point Likert scale, ranging from “strongly disagree” (0) to “strongly agree” (4). Positively worded items (n = 3) (e.g. ‘*In uncertain times*, *I usually expect the best*.’) were summed to calculate the optimism score and the negatively worded items (n = 3) to pessimism score (e.g. ‘*I rarely count on good things to happen to me*.’) [14]. The study analysed optimism and pessimism as empirically different constructs [35].

After harmonizing the LOT-R items, optimism and pessimism scales were scored as described previously [14]. In the case of only one missing value, the mean of the two available items replaced the missing value. Otherwise, the score was coded as missing. One participant lacked a pessimism scale score due to missing values, and hence we excluded this participant from the pessimism analyses.

#### Gestational age exposure measurements

Gestational age measured via ultrasound or date of the mother’s last menstrual period was obtained from medical records. Preterm birth was defined as ≤36+6 (weeks+days) of gestational age and term birth as ≥37+0 gestational age. We further categorized preterm birth to Early Preterm(<32+0, n = 193), Moderate Preterm (32+0 to 33+6, n = 57), and Late Preterm (34+0 to 36+6, n = 275) births.

#### Covariates and moderators

The variables considered as covariates included participant’s sex (women/men), as well as maternal mode of delivery(vaginal/cesarean section), smoking during pregnancy(yes/no), age at childbirth(years) and preeclampsia(yes/no), which were taken from medical records for all studies. The variables considered both as covariates and moderators included birth weight z-score, neurosensory impairment and parental education. Education level variables were harmonized according to the International Standard Classification of Education (ISCED) [36] and dichotomized into secondary education or less and tertiary education. Parental education was reported by the mother in childhood (ALSPAC, AYLS) and by the participant in adulthood(HeSVA) [36]. Birth weight was obtained from medical records and we calculated the birth weight z-score using INTERGROWTH-21 [37], the World Health Organization (WHO) international standardized growth curves which adjust for sex and gestational age. Neurosensory impairments were defined as yes if the participant had at least one of the following: cerebral palsy, intellectual disability (IQ<70), severe visual impairment or severe auditory deficit (n = 192) [4, 38–40]. Neurosensory impairments were assessed at the childhood clinical follow-ups in all three studies, with exception of IQ, which was assessed in adulthood in HeSVA [41].

### Statistical analysis

#### Synthesis method

To examine if preterm and term born young adults differed in optimism/pessimism and if optimism/pessimism varied according to the degree of prematurity, we employed a two-stage random-effects IPD meta-analysis. We fitted the linear regression models to the data from each cohort and then derived the random inverse-variance weighted pooled effects accounting for between-study heterogeneity tested using the Cochran’s Q statistic and quantified by the I^2^ value. Low heterogeneity was defined as an I^2^ value of 0% to 25%, moderate heterogeneity as an I^2^ of 25% to 75%, and high heterogeneity as an I^2^ of 75% to 100%.

Applying the same analytic approach, we examined if participant’s birth weight z-score, neurosensory impairments or parental education modified the developmental outcomes of those born preterm and at term. This was studied by including interaction terms of preterm birth vs term birth x birth weight z-score, preterm birth vs term birth x yes vs no neurosensory impairment and preterm birth vs term birth x parental education into the regression equations following the main effects of these variables.

We present the findings as adjusted for sex and for those covariates that were associated with dispositional optimism/pessimism. In preterm and term comparisons, we also conducted sensitivity analyses excluding participants with neurosensory impairment.

The study utilized Stata 15(StataCorp. 2017. Stata Statistical Software: Release 15. College Station, TX: StataCorp LLC.).

#### Power analyses

With alpha level 0.05 and 80% power we were able to detect differences between preterm born and term born adults and detect interactions that in effect size were small (N = 525 preterm adults and N = 4547 term born adults; Cohen’s d = 0.12 for the main effects and 0.48 for the interactions).

## Results

### Included studies

#### Study selection and IPD obtained

The electronic search of PubMed and Web of Science yielded 165 studies. Hand searching of grey literature and the preterm birth cohort networks yielded another 23 possible studies. The updated search yielded an addition 100 studies. After title and abstract review, 5 published studies underwent full-text review. None of the published studies from the electronic search met the inclusion criteria. Of the preterm birth networks’ cohorts with unpublished data, 3 studies met inclusion criteria (Fig 1). After contacting all three studies, each study provided the requested IPD for the analyses. The approved research proposal and data request for ALSPAC, #B3202 from the University of Bristol for the PremLife project can be found online [42]. Data collection for the ALSPAC cohort utilized the REDCap software [43, 44].

#### Quality of evidence assessment & IPD data integrity

As no published studies met the inclusion criteria, the quality of previously published evidence could not be assessed. To evaluate IPD data integrity, the datasets received from each cohort were checked for abnormalities, missing data, out-of-range, inconsistent items or for harmonization issues. No issues were identified when reviewing the IPD provided from any of the three cohort studies. We assessed the risk of bias in the IPD in duplicate (RR, KH) on the cohort level, using a modified version of the criteria set out by Crawford and colleagues [51] (S1 Table). Although common in adult follow ups of birth cohort studies, attrition was high, particularly in ALSPAC. We assessed the characteristics of participants lost to follow-up compared to those included in our analyses (S2 Table). Young adults who provided optimism/pessimism data in young adulthood in ALSPAC and AYLS were born more often at term, had higher birth weight z-scores, less often had neurosensory impairment, their mothers were older at childbirth, and their parents had higher education compared to those who did not provide optimism/pessimism data. In AYLS, non-participants were more often born via caesarean section than participants. In ALSPAC and HeSVA, participants’ mothers smoked less often during pregnancy and in all cohorts the participants were more often women. See S2 Table for detailed attrition data.

### Characteristics of the study population

The IPD-MA study population comprised three birth cohorts: ALSPAC; Born 1990–92, UK [35, 36], AYLS; Born 1985–86, Finland [37, 38], and the Helsinki Study of Very Low Birth Weight Adults (HeSVA; Born 1978–85, Finland) [4, 28, 41, 39–51]. Table 1 describes the characteristics of the study population by cohort. The combined study population (n = 5072) included 525 participants who were born preterm between 1978–1992 in Finland and the United Kingdom. All three cohorts have been described in detail in previous publications [35–37, 40].

**Table 1:** Characteristics of participants by cohort.

Characteristic		Preterm	Control	P for Preterm vs. Control
		N (%)/Mean (SD)	N (%)/Mean (SD)	
Participants	ALSPAC	200 (5.2)	3627 (94.8)	
	AYLS	167 (18.5)	753 (81.9)	
	HeSVA	158 (48.6)	167 (51.4)	
Sex, female	ALSPAC	131 (3.4)	2346 (61.3)	0.81
	AYLS	83 (9.0)	416 (45.2)	0.19
	HeSVA	91 (28.0)	102 (31.38)	0.52
Gestational Age, wk	ALSPAC	34.3 (2.5)	39.8 (1.3)	*<* .*0001*
	AYLS	34.3 (3.1)	40.0 (1.5)	*<* .*0001*
	HeSVA	29.2(2.5)	40.1 (1.3)	*<* .*0001*
Birthweight, g	ALSPAC	2382.1 (665.9)	34.55.8 (476.3)	*<* .*0001*
	AYLS	2384.7 (828.9)	3564.9 (574.2)	*<* .*0001*
	HeSVA	1122.0 (249.3)	3579.5 (525.8)	*<* .*0001*
Birthweight Z-Score	ALSPAC	0.3 (1.0)	0.4(1.0)	0.04
	AYLS	0.2 (1.1)	0.6 (1.1)	*<* .*0001*
	HeSVA	-0.4 (1.2)	0.6 (1.0)	*<* .*0001*
Neurosensory Impairment, yes	ALSPAC	9 (0.8)	151 (13.1)	0.76
	AYLS	5 (0.7)	8 (1.1)	0.05
	HeSVA	*NA* *	*NA* *	*NA* *
Maternal Age at Birth	ALSPAC	29.1 (4.8)	29.5 (4.6)	0.38
	AYLS	29.7 (5.6)	29.7 (5.2)	0.90
	HeSVA	29.8 (5.4)	29.7 (5.6)	0.80
Highest Parental Education, tertiary	ALSPAC	41 (20.5)	955 (26.3)	0.27
	AYLS	41 (24.6)	204 (27.1)	0.162
	HeSVA	44 (27.8)	70 (41.9)	.001
Smoked During Pregnancy, yes	ALSPAC	43 (1.2)	669 (20.2)	0.51
	AYLS	31 (4.1)	102 (13.6)	.001
	HeSVA	30 (9.2)	27 (8.3)	0.50
Delivery Mode, caesarean Section	ALSPAC	52 (2.2)	348 (14.9)	*<* .*0001*
	AYLS	86 (9.6)	143 (16.0)	*<* .*0001*
	HeSVA	77 (24.4)	17 (5.4)	*<* .*0001*
Preeclampsia, yes	ALSPAC	29 (0.8)	60 (1.6)	*<* .*0001*
	AYLS	*NA* *	*NA* *	*NA* *
	HeSVA	34 (11.9)	12 (4.2)	*<* .*0001*
Participant Age at Follow-up	ALSPAC	23.0 (0)	23.0 (0)	-
	AYLS	25.4 (0.7)	25.4 (0.7)	0.93
	HeSVA	22.5 (2.4)	22.5 (2.5)	0.93
Optimism Score	ALSPAC	6.9 (2.7)	7.0 (2.5)	0.82
	AYLS	7.9 (3.0)	8.0 (2.6)	0.84
	HeSVA	8.6 (2.7)	8.6 (2.7)	0.94
Pessimism Score	ALSPAC	5.7 (3.2)	5.4 (2.7)	0.15
	AYLS	4.2 (3.1)	4.0 (2.6)	0.28
	HeSVA	4.1 (2.8)	3.4 (2.8)	.001

*NA In order to protect the privacy of participants, this information is not provided.

†P-Value ≤0.05

‡ P-value < .0001

**Fig 2:**
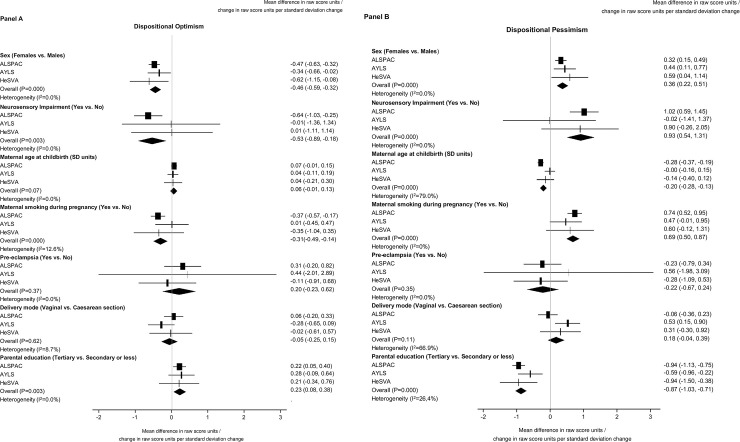
Association between covariates and dispositional optimism (A) and pessimism (B).

Among the combined datasets, mothers of preterm born participants were more likely than mothers of term borns to be primiparous, to have delivered via caesarean section, and to have had a multiple pregnancy or pre-eclampsia (Table 1). Fig 2 shows associations between the covariates and optimism (Panel A) and pessimism (Panel B). Men in comparison to women, and participants without neurosensory impairments, whose mothers did not smoke during pregnancy and whose parents had higher educational attainment, scored higher on optimism and lower on pessimism. Pessimism scores were also lower for those born to older mothers.

#### Optimism/Pessimism in preterm and term born young adults

**Fig 3:**
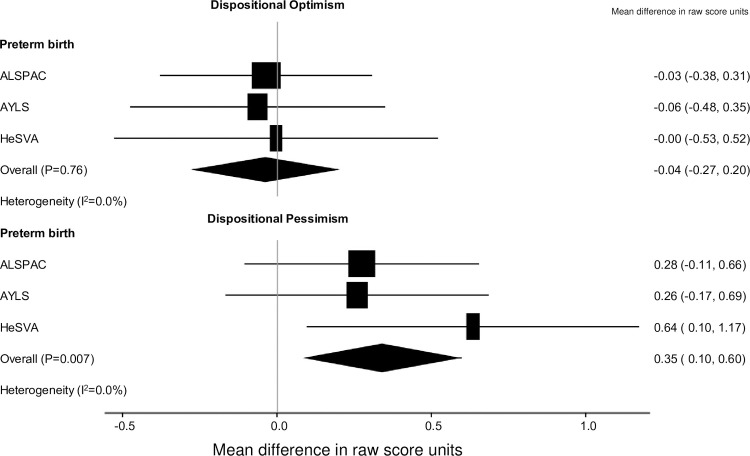
Dispositional optimism and pessimism and preterm birth.

In the pooled IPD-MA adjusted for participant’s sex, preterm born and term born young adults did not differ on optimism (Fig 3), but preterm born young adults scored higher on pessimism (Fig 3). Excluding 192 individuals with neurosensory impairments from the analyses did not alter the findings (optimism: Mean difference (MD) = -0.02 raw score units [95%CI -0.26, 0.22], p = 0.88; pessimism: MD = 0.29 raw score units [95%CI 0.04, 0.55], p = 0.03). After further adjustments for those covariates that were significantly associated with optimism/pessimism, namely maternal age at delivery, parental education and maternal smoking during pregnancy, the difference in pessimism between preterm born and term born adults became non-significant (MD = 0.17 raw score units [95%CI -0.11, 0.46], p = 0.23).

Degree of preterm birth was not associated with optimism (p>0.47 for unadjusted and adjusted models), but by each category decrease in preterm from term birth, pessimism scores increased by 0.14 raw score units [(95%CI 0.01, 0.27, p = 0.04). This increase in pessimism, became non-significant after covariate adjustment (p = 0.26).

#### Birth weight z-score, neurosensory impairments and parental education in preterm and term born adults

**Fig 4:**
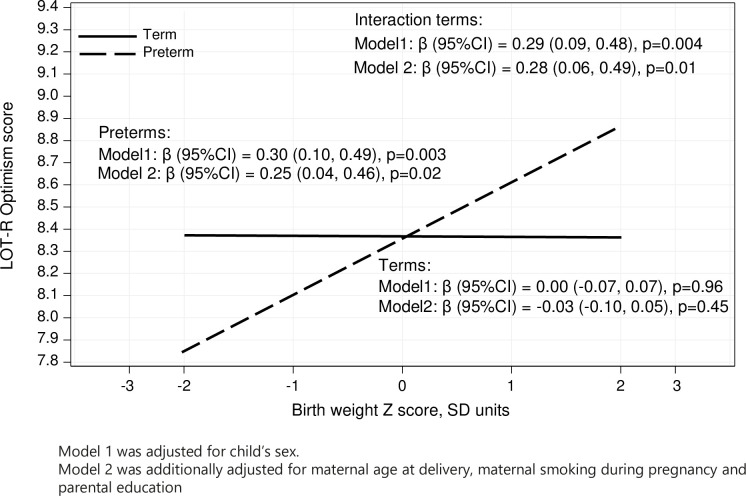
Preterm birth vs term birth and birth weight z-score interactions in the analysis of dispositional optimism.

In the pooled IPD-MA adjusted for participants’ sex, preterm born adults with higher birth weight z-score scored higher on optimism, while birth weight z-score was not associated with optimism in individuals born at term (Fig 4; p = 0.004 for preterm birth vs term birth x birth weight z-score interaction). The association between birth weight z-score and optimism among preterm born adults remained significant after covariate adjustments (Fig 4). There were no such interactions on pessimism, and the interactions between preterm birth vs term birth *x* yes vs no neurosensory impairment and preterm birth vs term birth *x* parental education on optimism and pessimism were not significant (p-values>0.66 for interactions).

## Discussion

Young adults born preterm and at term report no differences on optimism. This is in contrast to what we expected. However, we also found that preterm born adults scored higher on pessimism, although this difference was not significant after adjustments for covariates. A similar finding was observed according to the degree of prematurity in categorical gestational age groups. These findings help illuminate the similarities between preterm born and term born adults. Albeit surprisingly, given that preterm born individuals more often report neurocognitive, socioemotional and physical and mental health adversities [5, 10], which have individually been linked to lower optimism and higher pessimism [19, 21–24]. The lack of differences, however, may reflect that the adulthood follow-ups captured preterm born and term born young adults who fared better and would report less physical and mental health adversities than those who did not participate. While drop-out may offer some insight, all cohorts included in these analyses have individually reported that preterm birth to be associated with neurocognitive, socioemotional, and physical and mental health adversities in young adulthood [47, 52, 53].

However, in line with our hypothesis, we found that individuals born preterm with higher birth weight z-score had more optimistic life orientations. This finding aligns with previous literature where individuals born preterm and at Very Low Birth Weight(VLBW, <1500g), but whose birth weight was appropriate for gestational age(AGA; birth weight z-score within plus or minus 2 standard deviations or between the 10^th^ and 90^th^ percentile) report less often a depression diagnosis, use less often antidepressant medication, score lower on depressive symptoms [40] and report less problems in executive functioning [38] than individuals born preterm with VLBW and whose birth weight was small for gestational age (SGA; birth weight z-score less than minus 2 standard deviations or less than the 10^th^ percentile). Furthermore, young adults born very preterm who were not born with VLBW have better neurodevelopmental and physical health outcomes than preterms born with VLBW [10]. Notably, family environment may also play a role, as fathers of individuals born AGA VLBW preterm reported more supportive parenting than fathers of individuals born SGA VLBW preterm [47]. More supportive parenting has been related to higher optimism [54] in offspring. Hence, higher birthweight in preterm individuals may foster developmental trajectories and environments that promote more optimistic life orientations. Whether the more optimistic life orientation of the higher birthweight preterm born adult reflects their better mental and physical health or is associated with higher resilience in the face of adversity remains unknown.

Although we found that in the entire sample, those without neurosensory impairments and those with higher parental education had higher optimism and lower pessimism scores, these associations did not differ between preterm born and term born peers. Higher optimism and lower pessimism scores in those without neurosensory impairments is logical and suggests that the adversities imposed by neurosensory impairments are independent gestational age. In addition, previous studies have shown that higher parental education is associated with higher optimism and lower pessimism [54].

### Strengths & limitations

Our systematic review suggests that this is the first study to assess optimistic and pessimistic life orientations in preterm born and term born young adults using IPD meta-analysis with longitudinal cohort data from pregnancy into adulthood. The use of a singular outcome measure in all studies (LOT-R), reduces heterogeneity in study’s findings. The inclusion criteria that all cohorts included were from the modern neonatal medicine era, reduces confounding from different neonatal medicine practices, and curtails data attrition as more participants survived into adulthood. The collective statistical power of the IPD-MA, provides the largest assessment t of the optimism/pessimism in preterm born individuals to date. Study limitations include follow-up data attrition limiting the generalizability of the findings, as preterm born and term born individuals that did not participate in the follow-up may differ. While we were able to account for important covariates, we could not adjust for age at follow-up, as ALSPAC data precludes the sharing of birth dates and hence, calculation of age. However, all ALSPAC participants were followed-up at the age of 23 and the overall age range in the cohorts was 19 to 27.1 years.

## Conclusions and implications

Our IPD-MA showed that preterm born and term born young adults do not differ in dispositional optimism. Important childhood, maternal and familial covariates explained the difference observed in the higher pessimism scores. However, preterm born adults with higher birth weight z-scores were more optimistic than lower birth weight preterm born adults, suggesting that higher birth weights in preterm born infants may foster developmental trajectories that promote more optimistic life orientations. To better reflect the reality of preterm born individuals, positive developmental outcomes following preterm birth warrant further investigation.

## Supporting information

S1 Checklist (DOC)

S1 Table Risk of bias assessment. (PDF)

S2 Table Maternal and birth characteristics of young adults who did provide optimism/pessimism data in comparison to young adults who did not provide optimism/pessimism data in young adulthood. (PDF)

S1 File Search strategies. (PDF)
