# Supplementary material for: Optimism in adults born preterm: Systematic review and individual-participant-data meta-analysis
Source: PLoS One. 2021 Nov 18;16(11):e0259463. doi: 10.1371/journal.pone.0259463 (PMC8601551; doi:10.1371/journal.pone.0259463)
Supplement: S1 Table — (PDF) [file pone.0259463.s002.pdf]

Risk of Bias Assessment---Reviewer 1 (KH)

Individual Participant Data Meta-Analysis
Cohort Risk of Bias & Quality of Evidence Assessment Criteria
Adapted from Crawford et al. 2015

Research Question	ALSPAC	AYLS	HeSVA
Was the overall purpose of the study to derive or confirm associations between dispositional optimism and pessimism and gestational age?<br>Derive yes/no<br>Confirm yes/no	No	No	No
**METHODS**<br>**Setting/context**			
Describe the setting (primary care, hospital, GP practice, etc…)	All pregnant women residents in that part of the old administrative county of Avon were eligible to participate in ALSPAC if their estimated delivery date (EDD) fell between 1 April 1991 and 31 December 1992.	All infants born alive between March 15, 1985, and March 14, 1986 in Uusimaa Finland, who were hospitalized within 10 days of their birth<br><br>(n=1535). 658 infants born during the same period but not admitted to the neonatal ward were prospectively randomly	The original study cohort comprised 335 consecutive, very-low-birth-weight infants born between January 1978 and December 1985 who were discharged alive. A comparison group was selected from the records of all consecutive births at each birth hospital. For each very-low-birth-weight survivor, we selected the next available singleton infant born at term (gestational age, ≥37 weeks) of the same sex who was not small for gestational age (standard-

	N=14 541 pregnancies were recruited in 1990–92, increasing to 15 247 pregnancies by the age of 18 years	recruited from the 3 largest maternity hospitals in the study area; the neonate born after every second hospitalized infant was selected. Thereafter, the participants were invited to clinical follow-up visits at 5, 20, and 56 months and 24-26 years of age.	deviation score for birth weight, ≥−2). We invited the 255 very-low-birth-weight subjects and 314 subjects born at term who were living in the greater Helsinki area to participate in the study in young adulthood. There are several follow-ups in adulthood.
Who took the measurements/administered the questionnaires? (psychologist, research assistant nurse, etc…)	Questionnaires were self-reported. Gestational age and birth weight were derived from the hospital records.	Questionnaires were self-reported. Gestational age and birth weight were derived from the hospital records.	Questionnaires were self-reported. Gestational age and birth weight were derived from the hospital records.
Geographical location	The old administrative county of Avon/ Bristol area, UK	Uusimaa, Finland	Uusimaa, Finland
**Dates during which the study was conducted**			
Recruitment	1 April 1991 and 31 December 1992	March 15, 1985, and March 14, 1986	Original sample January 1978 and December 1985, Matched control group was identified 2004.

Examination	Not Applicable	Not Applicable	Not Applicable.
Follow-up	2013-2015	2009-2012	2007-2008
**Participants**			
**Describe the eligibility criteria**			
**Selection of patients**<br><br>Was the selection of patients conducted in such a way to avoid bias?<br><br>Yes: a consecutive or random sample /No: not a consecutive or random sample /Unclear: not enough information	Yes: a consecutive or random sample of patients was recruited.	Yes: a consecutive or random sample of patients was recruited	Yes: a consecutive or random sample of patients was recruited
**Timing of follow-up**	Yes: the follow-up was conducted in young adulthood	Yes: the follow-up was conducted in young adulthood	Yes: the follow-up was conducted in young adulthood

Was the timing of follow-up long enough i.e. young adulthood?<br><br>Yes/No/unclear			
**Replicating the tests**<br><br>Is there sufficient explanation of the follow up procedures and questionnaire use to permit their replication?<br><br>Yes/No/Unclear	Yes: the follow up procedures can be replicated from the explanation.<br><br>More detailed description of the original follow-up can be found from the cohort profile	Yes: the follow up procedures can be replicated from the explanation. More detailed description of the original follow-up can be found from the other referred articles.	Yes: the follow up procedures can be replicated from the explanation. More detailed description of the original follow-up can be found from the other referred articles.
**Blinding**<br><br>Were the investigators who collected the follow-up data blind to gestational age of the participants?<br><br>Yes/No/Unclear	Unclear: no information about the follow-up exists.<br><br>However, even though clear statement does not exist, all participants were invited with same protocol and outcome was self-reported.	Yes: the follow-up was conducted by investigators who were unaware of the gestational age of the participant	Yes: the follow-up was conducted by investigators who were unaware of the gestational age of the participant.
**Study size**<br>Has the study size been explained in detail?<br>Yes/No/Unclear	Yes: the study size has been explained in detail	Yes: the study size has been explained in detail	Yes: the study size has been explained in detail

**RESULTS**			
**Participant Follow-up**<br><br>Is a flow diagram available showing the numbers of individuals at all stages of the study, the numbers of potentially eligible patients, the numbers examined for eligibility, numbers included in the study, and numbers of completed follow-ups and outcomes)?<br>Yes/No/Unclear	Yes: a flow diagram showing the numbers of individuals at all stages of the study, the numbers potentially eligible, numbers examined for eligibility, numbers included in the study and numbers of completed follow-ups and outcomes exists<br><br>However, exact number at 23 year follow-up is not available in published papers.	Yes: a flow diagram showing the numbers of individuals at all stages of the study, the numbers potentially eligible, numbers examined for eligibility, numbers included in the study and numbers of completed follow-ups and outcomes exists	Yes: a flow diagram showing the numbers of individuals at all stages of the study, the numbers potentially eligible, numbers examined for eligibility, numbers included in the study and numbers of completed follow-ups and outcomes exists

Risk of Bias Assessment—Reviewer 2 (RR)

**Individual Participant Data Meta-Analysis<br>Cohort Risk of Bias & Quality of Evidence Assessment Criteria**<br>Adapted from Crawford et al. 2015			
**Research Question**	**ALSPAC**	**AYLS**	**HeSVA**
Was the overall purpose of the study to derive or confirm associations between dispositional optimism and pessimism and gestational age?<br>Derive yes/no<br>Confirm yes/no	No	No	No
**METHODS<br>Setting/context**			
Describe the setting (primary care, hospital, GP practice, etc…)	Initial recruitment Maternity clinics in Avon,<br><br>Follow up	Initial care provided in neonatal intensive care units and local labor wards.<br><br>Follow-up questionnaires—completed online.	Initial care provided at hospitals<br><br>Follow-up questionnaires—sent by mail.
Who took the measurements/administered the	Questionnaire by mail.	Internet based questionnaire-WEBprobol	Questionnaire by mail.

questionnaires? (psychologist, research assistant nurse, etc…)			
Geographical location	Avon region, United Kingdom	Uusimaa, Finland	Uusimaa, Finland
**Dates during which the study was conducted**			
Recruitment	1990-1992	1985-1986	1978-1985
Examination	Not applicable	Not applicable	Not applicable
Follow-up	2013-2015	2009-2012	2007-2008
**Participants**			
**Describe the eligibility criteria**	*"All pregnant women residents in that part of the old administrative county of Avon comprising the three Health Districts shown in Figure 2 were eligible toparticipate in ALSPAC if their estimated delivery date (EDD) fell between 1 April 1991 and 31December 1992 inclusive. Any resulting child from these pregnancies is considered eligible. The catchment area covered the three health*	***Preterm/VLBW participants***<br>*All infants born alive and admitted to neonatal wards in obstetric units or transferred to the Neonatal Intensive care unit between 15 March 1985 and 14 March 1986 in the study area.*	***VPT/VLBW Group Inclusion Criteria***<br>*All infants born at < 1500g discharged alive from the NICU at Children's Hospital in Helsinki between 1978 and 1985.*

	administration districts within the South-West Regional Health Authority that became the 'Bristol & District Health Authority'. This area (1991 total population 0.9 million) includes the City of Bristol (1991 population 0.5 million) and surrounding urban and rural areas, including towns, villages and farming communities; but excludes the area of Avon around the City of Bath. Pregnant women migrating into the catchment area were eligible up to the point of delivery; pregnant women originally resident in Avon but migrating out of the catchment area prior to delivery were excluded unless they had completed the questionnaire scheduled for the third trimester of pregnancy."(Boyd et al. 2012)	*No specific inclusion criteria related to gestational age or birth weight.*<br><br>***Control Group Inclusion Criteria***<br>*An infant born after every second hospitalized infant and without evidence of neonatal illness was identified from one of the three biggest maternity hospitals in the study area during the same period.*<br><br>*(Information From the RECAP preterm data-node)*	***Control Group Inclusion Criteria***<br>*Control subjects were group-matched by age, sex and birth hospital. They had to be singleton, term born and not SGA (Birth weight SDS not < -2.0 according to current Finnish criteria (Pihkala J 1989, Duodecim).*<br>*Control subjects were group-matched by age, sex and birth hospital. They had to be singleton, term born and not SGA (Birth weight SDS not < -2.0 according to current Finnish criteria (Pihkala J 1989, Duodecim).*<br><br>***VPT/VLBW Group Exclusion Criteria***<br>*Death before discharge from NICU. Lived more than 110 km away from Helsinki in 2004.*<br>***Control Group Exclusion Criteria***<br>*Lived more than 110 km away from Helsinki in 2004.*<br>*(Information From the RECAP preterm data-node)*

**Selection of patients**<br><br>Was the selection of patients conducted in such a way to avoid bias?<br><br>Yes: a consecutive or random sample /No: not a consecutive or random sample /Unclear: not enough information	It is difficult to say if this was a consecutive or not, as all pregnant women were eligible. And an opportunistic approach was taken to recruit all possible women within a fixed time period.<br><br>Of the 20248 eligible pregnancies, 14541were enrolled. Although, there is limited information on those who did not participate. ALSPAC has conducted a subsample record linkage study of 765 eligible non-participants/drop-outs. Overall, those who enrolled and participated in the 17 year follow up were more likely female and to have come from less deprived areas. No notable differences were observed in other baseline characteristics (*Letter to the Editor)*	Yes: a consecutive or random sample of patients was recruited	Yes: a consecutive or random sample of patients was recruited

**Timing of follow-up**<br><br>Was the timing of follow-up long enough i.e. young adulthood?<br><br>Yes/No/unclear	The follow up was conducted in young adulthood. However, in ALSPAC, we did not have the specific age of each of the participants, but generally at age 23.	Yes, the follow up was conducted in young adulthood.	Yes, the follow up was conducted in young adulthood.
**Replicating the tests**<br><br>Is there sufficient explanation of the follow up procedures and questionnaire use to permit their replication?<br><br>Yes/No/Unclear	ALSPAC Dataset: YPC_2a<br>Quest Questionnaire Follow-Up, Q1427_YPC.pdf (Section Wellbeing: C12 items a-j, page 20)<br>Participant Completed LOT-R Variables: YPC_0590-YPC_0599<br><br>Questionnaire provided detailed instructions to the participant which can be replicated.<br><br>Yes: the follow up procedures can be replicated from the explanation	Yes: the follow up procedures can be replicated from the explanation.	Yes: the follow up procedures can be replicated from the explanation

**Blinding**<br><br>Were the investigators who collected the follow-up data blind to gestational age of the participants?<br><br>Yes/No/Unclear	Whether the investigators who sent the questionnaire were blind to the gestational age status of the participants is unclear. However, as the follow-up was done via questionnaire. It is unlikely that the scores from the participants would be biased by the lack of blinding.	The investigators who send the questionnaires did not have access to the gestational age data.<br><br>The follow-up was done via questionnaire. It is unlikely that the scores from the participants would be biased by the lack of blinding.	The investigators who send the questionnaires did not have access to the gestational age data.<br><br>The follow-up was done via questionnaire. It is unlikely that the scores from the participants would be biased by the lack of blinding.
**Study size**<br><br>Has the study size been explained in detail?<br><br>Yes/No/Unclear	Yes: a sample-size is explained in sufficient detail.	Yes: a sample-size is explained in sufficient detail.	Yes: a sample-size is explained in sufficient detail.
**RESULTS**			
**Participant Follow-up**<br><br>Is a flow diagram available showing the numbers of individuals at all stages of the study, the numbers of potentially eligible patients, the numbers examined for eligibility, numbers included in the study, and numbers of completed	Yes: a flow diagram showing the numbers of individuals at all stages of the study, the numbers potentially eligible, numbers examined for eligibility, numbers	Yes: a flow diagram showing the numbers of individuals at all stages of the study, the numbers potentially eligible, numbers examined for eligibility, numbers included in the study and numbers of	Yes: a flow diagram showing the numbers of individuals at all stages of the study, the numbers potentially eligible, numbers examined for eligibility, numbers

follow-ups and outcomes)?<br>Yes/No/Unclear	included in the study and numbers of completed follow-ups and outcomes exists<br><br>Attrition rate was high.<br><br>Cohort profile by Boyd et al. 2012 provides a participant flow diagram. A later publication by Northstone et al. 2019 provides further detail.	completed follow-ups and outcomes exists<br><br>AYLS Participant flow diagram published by Kaseva et al. 2020.	included in the study and numbers of completed follow-ups and outcomes exists<br><br>HeSVA participant flow diagram published in Sipola-Leppänen et al. 2011.

*The above tables are modified versions of the table published by Crawford et al. 2015. The Copyright information for the for the original Crawford et al. publication is as follows:

Copyright © Queen's Printer and Controller of HMSO 2015. This work was produced by Crawford *et al.* under the terms of a commissioning contract issued by the Secretary of State for Health. This issue may be freely reproduced for the purposes of private research and study and extracts (or indeed, the full report) may be included in professional journals provided that suitable acknowledgement is made and the reproduction is not associated with any form of advertising. Applications for commercial reproduction should be addressed to: NIHR Journals Library, National Institute for Health Research, Evaluation, Trials and Studies Coordinating Centre, Alpha House, University of Southampton Science Park, Southampton SO16 7NS, UK.

References

Crawford F, Cezard G, Chappell FM, Murray GD, Price JF, Sheikh A, Simpson CR, Stansby GP, Young MJ. A systematic review and individual patient data meta-analysis of prognostic factors for foot ulceration in people with diabetes: the international research collaboration for the prediction of diabetic foot ulcerations (PODUS). Health Technol Assess. 2015 Jul;19(57):1-210. doi: 10.3310/hta19570. PMID: 26211920; PMCID: PMC4781379.

Boyd A, Golding J, Macleod J, Lawlor DA, Fraser A, Henderson J, Molloy L, Ness A, Ring S, Davey Smith G. Cohort Profile: the 'children of the 90s'--the index offspring of the Avon Longitudinal Study of Parents and Children. Int J Epidemiol. 2013 Feb;42(1):111-27. doi: 10.1093/ije/dys064. Epub 2012 Apr 16. PMID: 22507743; PMCID: PMC3600618.

Northstone K, Lewcock M, Groom A, Boyd A, Macleod J, Timpson N, Wells N. The Avon Longitudinal Study of Parents and Children (ALSPAC): an update on the enrolled sample of index children in 2019. Wellcome Open Res. 2019 Mar 14;4:51. doi: 10.12688/wellcomeopenres.15132.1. PMID: 31020050; PMCID: PMC6464058.

Kaseva, N., Vääräsmäki, M., Matinolli, HM. *et al.* Maternal pre-pregnancy overweight and gestational diabetes and dietary intakes among young adult offspring. *Nutr. Diabetes* **10,** 26 (2020). https://doi.org/10.1038/s41387-020-00129-w

Sipola-Leppänen M, Hovi P, Andersson S, Wehkalampi K, Vääräsmäki M, et al. (2011) Resting Energy Expenditure in Young Adults Born Preterm—The Helsinki Study of Very Low Birth Weight Adults. PLOS ONE 6(3): e17700. https://doi.org/10.1371/journal.pone.0017700
