# Supplementary material for: Optimism in adults born preterm: Systematic review and individual-participant-data meta-analysis
Source: PLoS One. 2021 Nov 18;16(11):e0259463. doi: 10.1371/journal.pone.0259463 (PMC8601551; doi:10.1371/journal.pone.0259463)
Supplement: S2 Table — (PDF) [file pone.0259463.s003.pdf]

	ALSPAC			AYLS			HeSVA		
	N	Mean (SD)/N (%)	P-value	N	Mean (SD)/N (%)	P-value	N	Mean (SD)/N (%)	P-value
Maternal age at birth (years)			<0.0001			0.004			0.67
Participants	3611	29.4 (4.5)		909	29.7 (4.9)		325	29.8 (4.9)	
Non-participants	8206	27.9 (4.9)		1232	29.2 (5.4)		245	28.7 (5.0)	
Tertiary parental education (yes)			<0.0001			0.001			0.71
Participants	3662	1009 (27.6%)		910	246 (27.0%)		323	114 (35.3%)	
Non-participants	8490	1344 (15.8%)		1191	249 (20.9%)		55	18 (32.7%)	
Smoking during pregnancy (yes)			<0.0001			0.24			0.02
Participants	3466	742 (21.4%)		748	133 (17.8%)		325	57 (17.5%)	
Non-participants	8878	3037 (34.2%)		141	31 (22.0%)		245	62 (25.3%)	
Mode of Delivery, Caesarean Section			0.275			<0.0001			0.024
Participants	394	(11.4%)		224	(24.4%)		94	(28.9%)	
Non-participants	1105	(9.1%)		420	(32.9%)		50	(20.4%)	
Preterm birth groups			<.0001			.007			.180
Early Preterm (<32+0 weeks+days)									
Participants	25	(0.2%)		27	1.2%		140	24.6%	
Non-participants	660	(4.5%)		65	3.0%		81	14.2%	
Moderate Preterm (32+0 to 33+6)									
Participants	20	(0.1%)		23	1.1%		12	2.1%	
Non-participants	96	(0.7%)		49	2.2%		11	1.9%	
Late Preterm (34+0 to 36+6)									
Participant	147	(1.0%)		114	5.2%		6	1.1%	
Non-participants	487	(3.3%)		201	9.2%		7	1.6%	
Female sex (yes)			<0.0001			<0.0001			0.008
Participants	3827	2477 (64.7%)		920	499 (54.2%)		325	193 (59.4%)	
Non-participants	10310	4347 (42.2%)		1268	498 (39.3%)		245	118 (48.2%)	
Birth weight (grams)			<0.0001			<0.0001			0.87
Participants	3775	3400.3 (529.4)		918	3351.5 (740.5)		325	2384.8 (1284.5)	
Non-participants	10115	3374.4 (598.7)		1254	3181.5 (847.7)		245	2651.9 (1296.6)	
Neuro impairment (yes)			<0.0001			0.01			0.15
Participants	1153	160 (13.9%)		760	13 (1.7%)		325	19 (5.9%)	
Non-participants	1696	446 (26.3%)		850	32 (3.8%)		245	8 (3.3%)	
