# Supplementary material for: Optimism in adults born preterm: Systematic review and individual-participant-data meta-analysis
Source: PLoS One. 2021 Nov 18;16(11):e0259463. doi: 10.1371/journal.pone.0259463 (PMC8601551; doi:10.1371/journal.pone.0259463)
Supplement: S1 File — (PDF) [file pone.0259463.s004.pdf]

Original Search Strategy

Pubmed: 165 articles
Beginning to 20.02.2019

((((((optimis* OR pessimis*)) AND (lot-r OR life orientation test revised)) AND (gestational age OR preterm OR prematur*)) AND (cohort OR case-control))) NOT (maternal AND (optimi* OR pessimi*))

Updated Search Performed 15-06-2021

PubMed: 54 articles

Search number	Query	Filters	Search Details	Results
1	(((("premature birth"[MeSH Terms]) OR (infant, very low birth weight[MeSH Terms]))) AND (("Life Orientation test"[Text Word])) OR (LOT-R[Text Word]))	from 2019 - 2021	((("premature birth"[MeSH Terms] OR "infant, very low birth weight"[MeSH Terms]) AND "Life Orientation test"[Text Word]) OR "LOT-R"[Text Word]) AND (2019:2021[pdat])	54

EBSCOhost/CLINHAL: 7 articles

6/15/2021 Print Search History: EBSCOhost

EBSCOhost

Tuesday, June 15, 2021 7:30:58 AM

#	Query	Limiters/Expanders	Last Run Via	Results
S1	TX "life orientation test" AND ("preterm birth" or "vlbw")	Limiters - Peer Reviewed; Research Article<br>Expanders - Apply equivalent subjects<br>Search modes - Boolean/Phrase	Interface - EBSCOhost Research Databases<br>Search Screen - Advanced Search<br>Database - CINAHL	7

ProQuest: 33 articles

SEARCH STRATEGY

Set No.	Searched for	Databases	Results
S1	("life orientation test" AND ("preterm birth" OR "VLBW" OR "very low birth weight" OR "gestational age")) AND (stype.exact("Scholarly Journals") AND PEER(yes))	Art, Design & Architecture Collection, Coronavirus Research Database, Early Modern Books, Ebook Central, EconLit, GeoRef, Literature Online, ProQuest Historical Newspapers: The Guardian and The Observer, ProQuest Historical Newspapers: The New York Times with Index, ProQuest Historical Newspapers: The Wall Street Journal, ProQuest Historical Newspapers: The Washington Post, PTSDpubs, Publicly Available Content Database, SciTech Premium Collection, Social Science Premium Collection	33

Web of Science: 6 articles

TOPIC: ("preterm birth" OR "very low birth weight" OR "VLBW" OR "gestational age") *AND* **TOPIC:** ("Life Orientation Test" OR LOT-R) *Indexes=SCI-EXPANDED, SSCI, A&HCI, CPCI-S, CPCI-SSH, BKCI-S, BKCI-SSH, ESCI Timespan=All years*
